# ﻿Redescription of *Microphysogobiotungtingensis* (Nichols, 1926) with the description of a new species of the genus (Cypriniformes, Gobionidae) from southern China

**DOI:** 10.3897/zookeys.1214.127061

**Published:** 2024-10-04

**Authors:** Zhi-Xian Sun, Wen-Qiao Tang, Ya-Hui Zhao

**Affiliations:** 1 Shanghai Universities Key Laboratory of Marine Animal Taxonomy and Evolution, Shanghai Ocean University, Shanghai, 201306, China Institute of Zoology, Chinese Academy of Sciences Beijing China; 2 Key Laboratory of Zoological Systematics and Evolution, Institute of Zoology, Chinese Academy of Sciences, Beijing, 100101, China Shanghai Ocean University Shanghai China

**Keywords:** East Asia, freshwater fish, Gobionidae, morphology, phylogeny, taxonomy

## Abstract

Although *Microphysogobiotungtingensis* (Nichols, 1926) has been treated valid since it was described, its morphology remains vague, especially when comparing it with another similar species, *M.elongatus* (Yao & Yang, 1977). In this study, the types of both species were examined and also compared with several lots of specimens from a wide geographical range: there is no significant difference in morphology between them. Additionally, molecular evidence supported by mitochondrial gene sequence also showed low genetic distance in between. Thus, it is suggested that *M.elongatus* is a junior synonym of *M.tungtingensis*. While revising these two species, a new species, *Microphysogobiopunctatus* sp. nov., was discovered that has a similar distribution with them both. However, it can be distinguished from its congeners by having a globular or oval shaped posterior air-bladder chamber which length 58.6%–82.8% of eye diameter; a narrow upper jaw cutting edge which less than half mouth width; a slender caudal peduncle with depth 34.6%–48.5% of length; and a six-branched-ray anal fin. This new species also has numerous small black spots on all fins which is also unique. The new species is morphologically and molecularly close to *M.bicolor* (Nichols, 1930).

## ﻿Introduction

The species of *Microphysogobio* Mori, 1934 are small gobionid fishes widely distributed in East Asia from northern Vietnam to eastern Russia, and eastern China is their main distribution region (Sun et al. 2022b; Fricke et al. 2024). They usually occur in the middle and upper reaches of the river system, preferring the sand and gravel mixed benthic habitats. Upon now, thirty-one *Microphysogobio* species were considered valid in the world, and approximately twenty-four of which were discovered in China (Sun et al. 2022a, 2022b; Fricke et al. 2024). The species in this genus has a three-lobe lower lip with developed papillae, horny sheaths on upper and lower jaw cutting margin and also a small posterior air-bladder chamber (Sun et al. 2021).

*Microphysogobiotungtingensis* was described by Nichols (1926a) based on one specimen collected near the lake Tungting, from Huping, Hunan (nowadays the Lake Dongting, Hubin Township in Yueyang City, Hunan Province), in the middle reaches of the Yangtze River basin. However, a more recent taxonomic study misidentified *M.tungtingensis* as a species that has only five branched anal-fin rays (vs six in holotype) based on three non-types (Luo et al. 1977). The subsequent taxonomic studies (Yue 1998; Huang et al. 2017, 2018; Sun and Zhao 2022) followed Luo et al. (1977). In fact, the branched anal-fin ray count in the genus *Microphysogobio* is conservative, and so it can be used as a key character in its taxonomy (Sun and Zhao 2022). Another species, *Microphysogobioelongatus*, originally described by Yao and Yang (in Luo et al. 1977) based on five syntypes collected in Guangxi Zhuang Autonomous Region, has a similar morphology with *M.tungtingensis*, and also a nearby distribution in the adjacent river basin (Luo et al. 1977; Yue 1998; Huang et al. 2017). This species, however, also has six branched anal-fin rays just as the holotype of *M.tungtingensis*. Therefore, a study needs to take a close look at both species and the validity of *M.elongatus*.

Although several new species were reported in recent studies (Huang et al. 2016, 2017, 2018; Sun et al. 2022b), the diversity of *Microphysogobio* is greatly underestimated. During the revision on *M.tungtingensis* and the so-called *M.elongatus*, we noticed several lots of specimens from Guangxi Zhuang Autonomous Region that were wrongly identified as *M.tungtingensis*. After thorough field investigations, with the morphological comparisons and molecular phylogenetic analyses, we describe them as a new species herein.

## ﻿Materials and methods

### ﻿Specimen collection, examination, and preservation

All examined specimens were collected by hand net, fish trap, or bought from the local markets. Detailed information on the specimens is listed in the section Comparative material. Specimens used for morphological study were fixed in 10% formalin solution for three days, followed by 70% ethanol alcohol for long-term preservation. Specimens used for molecular phylogenetic study were fixed in 95% ethanol. The holotype of the new species was deposited at the Institute of Zoology, Chinese Academy of Sciences, Beijing, China (**ASIZB**; abbreviation follows Leviton et al. 1985), paratypes were deposited at ASIZB, Institute of Hydrobiology, Chinese Academy of Sciences, Wuhan, China (**IHB**), and Laboratory of Ichthyology, Shanghai Ocean University, Shanghai, China (SHOU). The holotype of *M.tungtingensis* is stored at American Museum of Natural History, New York, the United States (**AMNH**). The syntypes of *M.elongatus* are stored at IHB. Other comparative materials are also deposited at ASIZB, IHB, and SHOU.

### ﻿Morphological study

Measurements were taken point-to-point with a digital caliper to 0.01 mm on the left side of the specimens, and counts were also made on the left side of specimens. In order to make a more accurate measurement for lips structure, photographs of the lip papillae system were taken and images were analyzed with ImageJ v. 1.52 software. Once the scale was settled, the distance between two points was measured with a straight line (Lunghi et al. 2019). Counts and measurements followed Sun et al. (2021). In order to get a general perception on external morphologic differences, 30 different measurable traits, log_10_-standardized to eliminate the allometries, were input into Past v. 4.03 (Hammer et al. 2001) for principal component analysis (PCA).

### ﻿Molecular phylogenetic analyses

Molecular phylogenetic studies were based on the mitochondrial Cytochrome-*b* (Cyt-*b*) sequences. DNA was extracted from the pelvic fin on the right side of the fish. Cyt-*b* was amplified using the primers cytbF1 (5’-TGACTTGAAGAACCACCGTTGTA-3’) and cytbR1 (5’-CGATCTTCGGATTACAAGACCGATG-3’) following Huang et al. (2016). Sequencing reactions were performed according to the operating instructions of BigDye Terminator v. 3.1 (BDT), with 1 μL of primer (3.2 pmol/μL), 1 μL of template DNA, 2 μL of BigDye® Terminator v. 3.1, and 6 μL of double distilled water (dd H2O) for a total reaction volume of 10 μL. The thermo-cycling conditions were initial denaturation for 2 min at 96 °C, denaturation for 10 s at 96 °C, annealing for 10 s at 50 °C, and extension for 1 min at 60 °C. After 30 cycles, a final extension was performed at 60 °C for 3 min and the polymerase chain reaction (PCR) products were preserved at 4 °C. Sequencing was conducted by Beijing TsingKe Biotech Co., Ltd. (China).

The sequencing results were assembled using SeqMan II, and other sequences were acquired from the NCBI database. The voucher ID of each individual and GenBank accession No. are given in Table 1. Forty-three Cyt-*b* sequences of *Microphysogobio* species were included in the molecular phylogenetic analyses. *Pseudogobioguilinensis* was used as outgroup. Nucleotide sequence alignment was conducted using MEGA v. 6.0 (Tamura et al. 2013) with ClustalW. ModelFinder (Kalyaanamoorthy et al. 2017) was used to select the best-fit model using Bayesian information criterion (BIC). The Bayesian inference (BI) phylogenies were inferred using MrBayes v. 3.2.6 (Ronquist et al. 2012) under the HKY+F+G4 model (two parallel runs, 1 000 000 generations), with the initial 25% of sampled data discarded as burn-in. Maximum likelihood (ML) phylogenetic analysis was conducted using MEGA 6.0 under the HKY+G+I model (10 000 bootstrap replications). Trees were visualized by using TVBOT (https://www.chiplot.online/tvbot.html; Xie et al. 2023). Each sequence was labeled with its own taxonomic nomenclature and the evolutionary divergence of sequence pairs between and within groups (i.e., species) was estimated using the Kimura 2-parameter model (Kimura 1980). In addition, two independent methods, i.e., Poisson tree process (PTP) and assemble species by automatic partitioning (ASAP), which rely on different operational criteria, were applied to infer molecular species delineation for *Microphysogobio* (Kapli et al. 2017; Puillandre et al. 2020). The rooted phylogenetic trees (both BI and ML) were uploaded to the PTP online server (http://species.h-its.org/ptp/). Aligned sequences were uploaded to the ASAP online server using the Jukes-Cantor (JC69) model (https://bioinfo.mnhn.fr/abi/public/asap/). The delineation results were visualized on the trees, respectively.

**Table 1.** Voucher code, sampling localities, haplotypes, and accession numbers of *Microphysogobio* species and outgroup for molecular analyses.

Voucher Code	Species	Locality	Drainage	Haplotype	Accession no.	Source
ASIZB 240450	*Microphysogobiopunctatus* sp. nov.	Lingchuan County, Guangxi Zhuang Aut. Reg. China	R. Lijiang, Pearl River Basin	H1	–	This study
ASIZB 240452	*M.punctatus* sp. nov.	Lingchuan County, Guangxi Zhuang Aut. Reg. China	R. Lijiang, Pearl River Basin	H2	–	This study
ASIZB 240451	*M.punctatus* sp. nov.	Lingchuan County, Guangxi Zhuang Aut. Reg. China	R. Lijiang, Pearl River Basin	H3	–	This study
ASIZB 240554	*M.punctatus* sp. nov.	Yongfu County, Guangxi Zhuang Aut. Reg. China	R. Luoqingjiang, Pearl River Basin	H4	–	This study
ASIZB 240555	*M.punctatus* sp. nov.	Yongfu County, Guangxi Zhuang Aut. Reg. China	R. Luoqingjiang, Pearl River Basin	H5	–	This study
ASIZB 240539	*M.punctatus* sp. nov.	Yongfu County, Guangxi Zhuang Aut. Reg. China	R. Luoqingjiang, Pearl River Basin	H6	–	This study
ASIZB 240548	*M.punctatus* sp. nov.	Guanyang County, Guangxi Zhuang Aut. Reg. China	R. Xiangjiang, middle Yangtze River Basin	H7	–	This study
ASIZB 240549	*M.punctatus* sp. nov.	Guanyang County, Guangxi Zhuang Aut. Reg. China	R. Xiangjiang, middle Yangtze River Basin	H8	–	This study
ASIZB 240543	*M.punctatus* sp. nov.	Guanyang County, Guangxi Zhuang Aut. Reg. China	R. Xiangjiang, middle Yangtze River Basin	H9	–	This study
ASIZB 240544	*M.punctatus* sp. nov.	Guanyang County, Guangxi Zhuang Aut. Reg. China	R. Xiangjiang, middle Yangtze River Basin	H8	–	This study
ASIZB 220619	*M.bicolor*	Yanshan County, Jiangxi Prov. China	R. Xinjiang, middle Yangtze River Basin	H10	OM803135	Sun and Zhao 2022
ASIZB 220620	*M.bicolor*	Yanshan County, Jiangxi Prov. China	R. Xinjiang, middle Yangtze River Basin	H11	OM803136	Sun and Zhao 2022
ASIZB 220630	*M.bicolor*	Wuyuan County, Jiangxi Prov. China	R. Raohe, middle Yangtze River Basin	H12	OM803140	Sun and Zhao 2022
ASIZB 220646	*M.bicolor*	Wuyuan County, Jiangxi Prov. China	R. Raohe, middle Yangtze River Basin	H13	OM803141	Sun and Zhao 2022
MYUVN1	*M.yunnanensis*	Dien Bien Prov. Vietamn	R. Lixianjiang, Red River Basin	H14	MK133329	Huang et al. 2018
MLURJ1	*M.luhensis*	Luhe County, Guangdong Prov. China	Rongjiang River Basin	H15	KT877355	Huang et al. 2018
MKAND1	*M.kachekensis*	Nankai Town, Hainan Prov. China	Nandujiang River Baisn	H16	KM999930	Huang et al. 2016
ASIZB 240446	*M.tungtingensis*	Xiangtan City, Hunan Prov. China	R. Xiangjiang, middle Yangtze River Basin	H17	–	This study
ASIZB 240445	*M.tungtingensis*	Xiangtan City, Hunan Prov. China	R. Xiangjiang, middle Yangtze River Basin	H18	–	This study
ASIZB 240430	*M.tungtingensis*	Yuelu District, Hunan Prov. China	R. Xiangjiang, middle Yangtze River Basin	H19	–	This study
ASIZB 240431	*M.tungtingensis*	Yuelu District, Hunan Prov. China	R. Xiangjiang, middle Yangtze River Basin	H20	–	This study
ASIZB 240462	*M.elongatus*	Yongfu County, Guangxi Zhuang Aut. Reg. China	R. Luoqingjiang, Pearl River Basin	H17	–	This study
ASIZB 240461	*M.elongatus*	Yongfu County, Guangxi Zhuang Aut. Reg. China	R. Luoqingjiang, Pearl River Basin	H17	–	This study
ASIZB 240463	*M.elongatus*	Yongfu County, Guangxi Zhuang Aut. Reg. China	R. Luoqingjiang, Pearl River Basin	H21	–	This study
ASIZB 240553	*M.elongatus*	Yangshuo County, Guangxi Zhuang Aut. Reg. China	R. Lijiang, Pearl River Basin	H17	–	This study
MELQZ1	*M.elongatus*	Quanzhou County, Guangxi Zhuang Aut. Reg. China	R. Xiangjiang, middle Yangtze River Basin	H17	KU356199	Huang et al. 2017
MFUMJ1	*M.fukiensis*	Shaowu City, Fujian Prov. China	R. Futunxi, Minjiang River Basin	H22	KM999927	Huang et al. 2016
MFUMJ2	*M.fukiensis*	Shaowu City, Fujian Prov. China	R. Futunxi, Minjiang River Basin	H23	KM999928	Huang et al. 2016
MFUMJ3	*M.fukiensis*	Xinquan Town, Fujian Prov. China	R. Tingjiang, Hanjiang River Basin	H24	KM999929	Huang et al. 2016
ASIZB 220771	*M.fukiensis*	Guangze County, Fujian Prov. China	R. Futunxi, Minjiang River Basin	H25	OM803150	Sun and Zhao 2022
ASIZB 220772	*M.fukiensis*	Guangze County, Fujian Prov. China	R. Futunxi, Minjiang River Basin	H26	OM803151	Sun and Zhao 2022
ASIZB 220802	*M.fukiensis*	Wuyishan City, Fujian Prov. China	R. Jianxi, Minjiang River Basin	H27	OM803152	Sun and Zhao 2022
ASIZB 220803	*M.fukiensis*	Wuyishan City, Fujian Prov. China	R. Jianxi, Minjiang River Basin	H27	OM803153	Sun and Zhao 2022
20170925BB05	*M.kiatingensis*	Chengdu City, Sichuan Prov. China	Upper Yangtze River Basin	H28	MG797640	Zou et al. 2018
MZHGC1	*M.zhangi*	Gongcheng County, Guangxi Zhuang Aut. Reg. China	R. Gongchenghe, Pearl River Basin	H29	KT877354	Huang et al. 2017
MZHGL1	*M.zhangi*	Guilin City, Guangxi Zhuang Aut. Reg. China	R. Lijiang, Pearl River Basin	H30	KU356194	Huang et al. 2017
MZHQZ1	*M.zhangi*	Quanzhou County, Guangxi Zhuang Aut. Reg. China	R. Xiangjiang, middle Yangtze River Basin	H31	KU356196	Huang et al. 2017
ASIZB 220682	*M.zhangi*	Yanshan County, Jiangxi Prov. China	R. Xinjiang, middle Yangtze River Basin	H32	OM803145	Sun and Zhao 2022
ASIZB 220715	*M.zhangi*	Wuyuan County, Jiangxi Prov. China	R. Raohe, middle Yangtze River Basin	H33	OM803148	Sun and Zhao 2022
MABC01	*M.alticorpus*	Chiayi County, Taiwan Prov. China	Bazhang River Basin	H34	KM031524	Jean et al. 2014
MXIML1	*M.xianyouensis*	Xianyou County, Fujian Prov. China	Mulanxi River Basin	H35	KM999931	Huang et al. 2016
ASIZB 220826	*M.oujiangensis*	Jinyun County, Zhejiang Prov. China	R. Panxi, Oujiang River Basin	H36	OM803130	Sun et al. 2022b
MBDH01	*M.brevirostris*	Taoyuan City, Taiwan Prov. China	R. Dahan, Tamshui River Basin	H37	KP168487	Chang et al. 2016
**Outgroup**						
PG-YS01	*Pseudogobioguilinensis*	Yangshuo County, Guangxi Zhuang Aut. Reg. China	R. Lijiang, Pearl River Basin		KX096699	He et al. 2017

## ﻿Results

### ﻿Taxonomic account

#### ﻿ Microphysogobio tungtingensis

Taxon classificationAnimaliaCypriniformesCyprinidae

(Nichols, 1926)

D8B343C3-CD0E-58DA-9CCE-728E21758AB1

**Figure 1.**
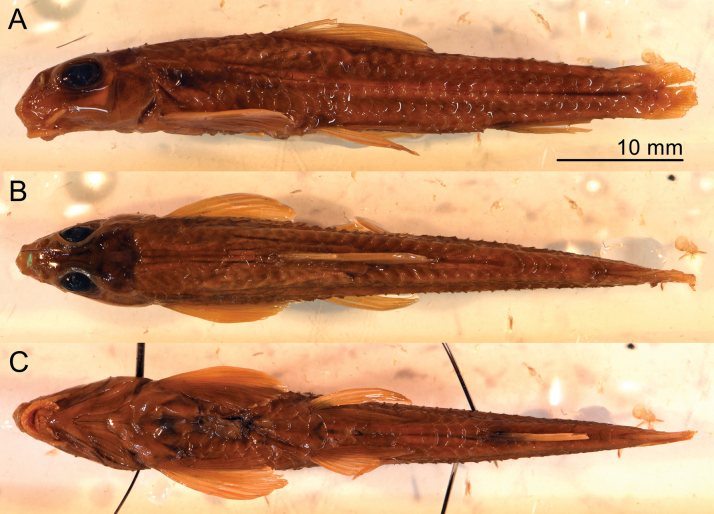
*Microphysogobiotungtingensis*, holotype, AMNH 8447, 51.4 mm SL **A** lateral view **B** dorsal view **C** ventral view; photographed by Dr. Thomas Vigliotta.

**Figure 2.**
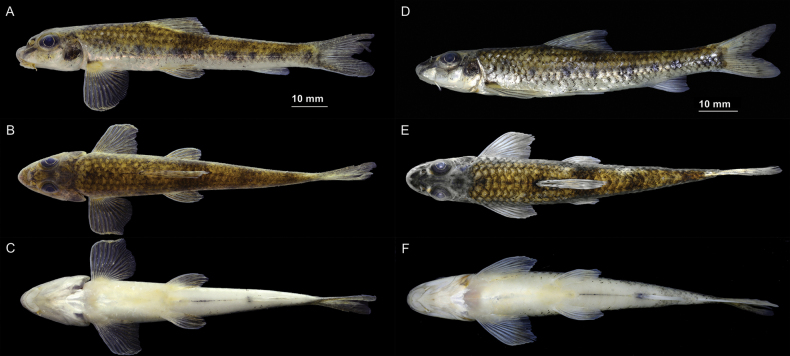
Lateral (**A**), dorsal (**B**), and ventral (**C**) views of *Microphysogobiotungtingensis*, ASIZB 240560, 81.8 mm SL, from the Jinjianghe River, a tributary of the Xiangjiang River, in Yuelu District, Changsha City, Hunan Province; Lateral (**D**), dorsal (**E**), and ventral (**F**) views of *M.elongatus* (= *M.tungtingensis*), ASIZB 240426, 72.3 mm SL, from the Lijiang River, the Pearl River Basin, in Pingle County, Guilin City, Guangxi Autonomous Region.

Figs 1 , 2 ; Table 2

Pseudogobio tungtingensis Nichols, 1926: 4 (original description); Nichols (1943): 180.
Microphysogobio tungtingensis : Bănărescu and Nalbant (1966): 201 (partim: Huping on lake Tungting, Hunan).
Abbottina elongata : Yao and Yang in Luo et al. (1977): 524.
Microphysogobio elongatus : Yue (1998): 362 (partim: Guilin, Liujiang, and Ningming in Guangxi); Zhang and Zhao (2016): 74; Xiao and Lan (2023): 154.
Microphysogobio kiatingensis : Xiao and Lan (2023): 152.

##### Material examined.

***Holotype*.** • AMNH 8447, 51.3 mm standard length (SL); Huping (= Hubin), Tungting Lake (= Lake Dongting), Hunan (= Hunan Province), China; collected by Clifford H. Pope; 1921.

##### Additional material examined.

• ASIZB 240436–44, 9 specimens, 50.8–73.0 mm SL; Changsha City, Hunan Province, from the confluence of the Jinjianghe River and the Xiangjiang River (28.13403751°N, 112.95238278°E, 29 m a.s.l.); collected by Mr. Tao Jiang, 22 October 2022 • ASIZB 240430–2, 3 specimens, 58.9–69.4 mm SL; Ximaowu Village, Yisuhe Township, Xiangtan County, Xiangtan City, Hunan Province, from the Juanshui River (27.73808121°N, 112.88006622°E, 36 m a.s.l.); collected by Mr. Tao Jiang; 7 December 2022 • ASIZB 240386–92, 7 specimens, 70.9–84.7 mm SL; Longxia Village, Hukou Township, Chaling County, Zhuzhou City, Hunan Province, from the Mishui River (26.56112501°N, 113.60761060°E, 144 m a.s.l.); collected by Zhixian Sun; 1 October 2020 • ASIZB 240395–404, 10 specimens, 54.6–74.5 mm SL; Yuelu District, Changsha City, Hunan Province, from the Jinjianghe River (28.12234231°N, 112.90897867°E, 29 m a.s.l.); collected by Mr. Tao Jiang; 8–17 June 2023 • ASIZB 240445–9, 5 specimens, 43.1–59.4 mm SL; Yuelu District, Changsha City, Hunan Province, from the Jinjianghe River (28.12234231°N, 112.90897867°E, 29 m a.s.l.); collected by Mr. Tao Jiang; 25 November 2023 • IHB 587820, 1 specimen, 65.9 mm SL; Guilin City, Guangxi Zhuang Autonomous Region, from the Lijiang River; July 1958 • IHB 587249, 587254, 587876, 3 specimens, 74.3–79.0 mm SL; Liujiang District, Liuzhou City, Guangxi Zhuang Autonomous Region, from the Liujiang River; July 1958 • IHB 587915, 1 specimen, 64.2 mm SL; Ningming County, Chongzuo City, Guangxi Zhuang Autonomous Region, from the Mingjiang River • ASIZB 240340–5, 240461–77, 23 specimens, 61.3–75.3 mm SL; Shangtai Village, Yongfu Township, Yongfu County, Guilin City, Guangxi Zhuang Autonomous Region, from the Xihe River (25.01610083°N, 109.98018869°E, 140 m a.s.l.); collected by Zhixian Sun, Chen Tian and Dong Sheng; 2 January 2024 • ASIZB 185300–5, 6 specimens, 59.4–81.4 mm SL; Jiangzhou District, Chongzuo City, Guangxi Zhuang Autonomous Region, from the Zuojiang River; 14 March 2003 • ASIZB 185204–5, 2 specimens, 59.4–61.3 mm SL; Sanjiang Dong Autonomous County, Liuzhou City, Guangxi Zhuang Autonomous Region, from the Liujiang River; 8 March 2003 • ASIZB 240393–4, 2 specimens, 72.9–74.9 mm SL; Yangshuo County, Guilin City, Guangxi Zhuang Autonomous Region, from the Lijiang River; collected by Zhixian Sun; 8 July 2019 • ASIZB 240427–8, 2 specimens, 54.6–67.9 mm SL; Pingle County, Guilin City, Guangxi Zhuang Autonomous Region, from the Lijiang River; collected by Zhixian Sun; 16 January 2020 • ASIZB 240426, 1 specimen, 72.3 mm SL; Pingle County, Guilin City, Guangxi Zhuang Autonomous Region, from the Lijiang River; collected by Zhixian Sun; 10 January 2021.

**Table 2.** Morphometric measurements of *Microphysogobiotungtingensis* and *M.elongatus*. Numbers in the brackets indicate number of specimens.

Characters	*Microphysogobiotungtingensis* (*n* = 35)				*Microphysogobioelongatus* (*n* = 41)				
	Holotype	Holotype + other specimens			Syntypes (*n* = 5)		Syntypes + other specimens		
		Range	Mean	SD	Range	Mean	Range	Mean	SD
Branched dorsal-fin rays	7	7	7		7	7	7	7	
Branched anal-fin rays	6	6	6		6	6	6	6	
Branched pectoral-fin rays	13	11–13	12		11–12	12	11–12	12	
Branched pelvic-fin rays	7	7	7		7	7	7	7	
Lateral line scales	39	36–39	37		37–38	37	36–38	37	
Scales above lateral line	3.5	3.5	3.5		3.5	3.5	3.5	3.5	
Scales below lateral line	2	1.5–2	2		1.5–2	2	1–2	2	
Pre-dorsal scales	9	8–10	9		9–10	10	8–11	10	
Circumpeduncular scales	12	12	12		12	12	12	12	
Standard Length (mm)	51.3	43.1–84.7			64.2–79.0	72.1	54.6–81.4		
**As percentage of SL**									
Body depth	15.7	14.7–20.1	17.3	1.5	15.5–17.4	16.3	15.5–19.3	17.1	0.9
Head length	22.2	21.4–26.1	23.6	1.1	22.2–24.5	23.1	21.5–25.3	23.5	0.9
Dorsal-fin length	22.9	20.3–24.3	23.0	1.0	20.7–23.3	22.3	20.7–25.7	23.6	1
Dorsal-fin base length	14.8	11.6–14.8	13.3	0.7	12.5–13.4	13	12.0–14.1	13.1	0.5
Pectoral-fin length	21.2	19.0–23.7	21.2	1.2	20.4–23.6	22	19.4–25.0	22.4	1.3
Pectoral-fin base length	4.6	4.6–6.3	5.5	0.4	5.1–5.7	5.4	4.2–6.2	5.3	0.5
Pelvic-fin length	16.8	14.1–19.5	16.6	1.0	15.9–17.3	16.7	15.9–19.7	17.5	0.9
Pelvic-fin base length	3.5	3.3–4.9	4.1	0.4	3.4–4.0	3.7	3.3–4.7	4	0.4
Anal-fin length	15.0	12.7–17.7	15.0	1.1	13.5–16.0	14.5	13.5–18.1	15.7	1.1
Anal-fin base length	7.6	3.6–8.6	7.0	0.8	5.7–7.3	6.5	5.7–8.9	7	0.7
Pre-dorsal length	42.5	41.0–46.2	43.8	1.3	42.2–43.5	42.8	40.3–45.6	43.4	1.1
Pre-pectoral length	23.7	22.5–26.4	24.8	0.9	24.0–25.5	24.4	22.9–26.4	24.7	1
Pre-pelvic length	47.0	47.0–51.9	48.3	1.2	47.1–49.3	48.3	46.4–51.4	48.8	1.2
Pre-anal length	76.5	74.6–81.2	77.8	1.5	74.2–80.1	77.4	74.2–80.2	77.5	1.4
Caudal peduncle length	17.1	13.2–18.2	15.5	1.1	14.8–17.0	15.5	14.3–18.1	16	0.9
Caudal peduncle depth	7.7	7.4–9.4	8.3	0.4	7.8–8.4	8.1	7.6–9.3	8.4	0.4
Head Length (mm)	11.4	9.9–19.4			16.7	15.0–18.2	12.2–18.9		
**As percentage of HL**									
Head depth	57.9	46.9–60.7	54.9	3.3	53.7–56.9	55.2	48.8–59.1	54.6	2.3
Head width	65.8	49.5–65.8	59.9	3.2	52.7–58.8	55.5	52.7–62.9	58.3	2.4
Eye diameter	30.9	29.3–37.3	33.2	1.6	32.2–35.1	33.7	30.7–38.9	34.9	2.2
Interorbital width	20.8	13.4–25.2	19.3	2.9	18.5–23.2	21	16.6–23.2	19.8	1.7
Snout length	38.6	33.4–45.8	38.9	2.9	34.3–40.2	37.3	30.4–40.2	36	2
Anterior papillae length	28.4	22.3–32.0	27.3 (31)	2.6	26.4–36.9	31	25.2–36.9	28.6	2.6
Anterior papillae width	22.7	17.4–36.3	26.9 (31)	4.5	22.2–28.9	24.6	14.3–28.9	21.5	3.6
Central anterior papillae width	5.7	5.1–8.5	6.7 (31)	0.9	5.3–7.3	6.4	4.5–9.2	6.9	1.3
Upper jaw cutting edge width	7.8	4.7–9.1	6.7 (31)	1.3	6.2–7.1	6.6 (3)	4.4–7.7	5.9 (37)	0.8
Medial pad width	10.2	7.5–12.1	10.0 (31)	1.0	8.8–13.1	11.6	7.0–13.4	10.8	1.2
Mouth depth	15.1	14.7–22.7	17.9 (31)	2.0	17.7–21.2	19.1	15.2–21.2	18	1.3
Mouth width	18.0	18.0–28.6	22.4 (31)	2.7	18.8–24.2	20.9	16.9–27.2	21.2	2.5
Barbel length	20.6	12.0–23.2	16.5 (31)	2.9	15.3–24.8	18.3	14.7–24.8	17.7	2.2

##### Diagnosis.

Posterior chamber of air-bladder small, elongated oval shaped, length 9.2%–11.8% of head length, and 30.4%–34.9% of eye diameter; upper jaw cutting-edge narrow, width less than half mouth width; lateral-line scales 36–39 (mode and mean 37); circumpeduncular scales 12; branched anal-fin rays 6; midventral region of body scaleless only before pectoral-fin base end.

##### Redescription.

Body elongated, thoracic region flattened, abdomen rounded, caudal peduncle short, compressed laterally. Dorsal body profile rising from nostrils to dorsal-fin origin, dropping along dorsal-fin base, then gradually sloping to caudal-fin base. Maximum body depth at dorsal-fin origin, body depth 14.7%–20.1% of standard length. Head elongated, length larger than body depth; snout blunt, with concavity on top of snout before nostrils; eye diameter 29.3%–38.9% of head length, located at dorsal half of head; interorbital region flattened, width smaller than eye diameter (28.6%–50.5% of eye diameter). Anus positioned at anterior one-third of distance from pelvic-fin insertion to anal-fin origin.

**Figure 3.**
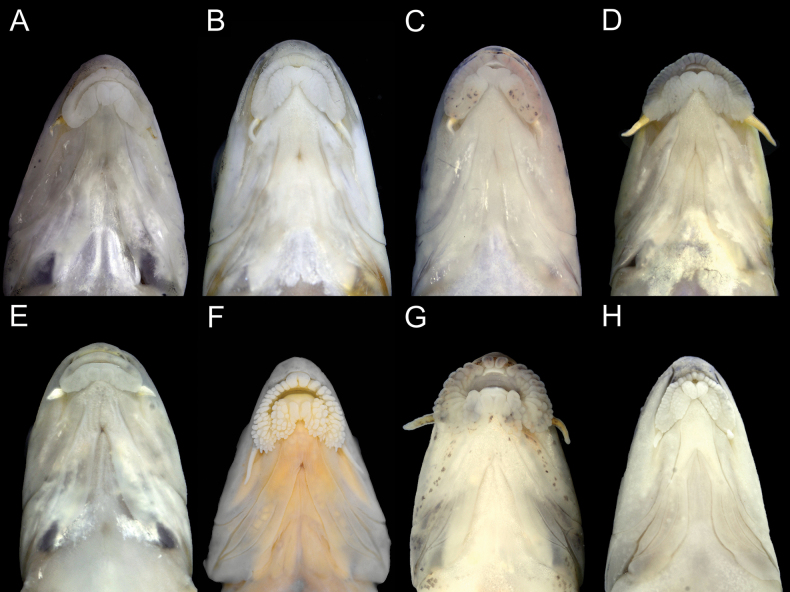
Lip papillae patterns of *Microphysogobio* species: **A***M.tungtingensis*, ASIZB 240560 **B***M.elongatus* (= *M.tungtingensis*), ASIZB 240426 **C***M.punctatus* sp. nov., holotype, ASIZB 240329 **D***M.punctatus* sp. nov., ASIZB 240300 **E***M.microstomus*, SHOU 20231209017 **F***M.zhangi*, holotype, ASIZB 204677 **G***M.vietnamica*, ASIZB 240571 **H***M.bicolor*, ASZIB 220638.

Mouth horseshoe-shaped and inferior, with one pair of maxillary barbels rooted at extremity of upper lip, barbel length shorter than eye diameter (35.2%–71.7% of eye diameter); upper and lower jaws with thin horny sheaths on cutting margins, upper jaw cutting edge width smaller than half mouth width (17.8%–43.2% of mouth width). Lips thick, well developed, with pearl-like papillae; central portion of anterior papillae arranged in one row with 2–4 well developed papillae, size slightly larger than other lateral side papillae; lateral portions of anterior papillae in several rows; medial pad on lower lip heart-shaped, or bisected into two oval-shape pads, rarely grooved on surface; lateral lobes on lower lip covered with multiple developed papillae, posteriorly disconnected from each other behind medial pad and laterally connected with upper lip anterior papillae at mouth corner (Fig. 3A, B).

Body covered with moderately small cycloid scales. Lateral line complete, almost straight in lateral center, slightly bent down under dorsal origin. Lateral line scales 36 (20 specimens), 37 (37), 38 (17), 39 (2); scales above lateral line 3.5 (76); scales below lateral line 1 (1), 1.5 (25), 2 (50); pre-dorsal scales 8 (7), 9 (33), 10 (35), 11 (1); circumpeduncular scales 12 (76). Midventral region of body scaleless only before pectoral-fin base end.

Dorsal fin with three unbranched and seven (76 specimens) branched rays; distal margin slightly concave, origin nearer to snout than caudal-fin base. Pectoral fin with one unbranched and 11 (12), 12 (50), 13 (14) branched rays; tip of adpressed not reaching anterior margin of pelvic-fin base. Pelvic fin with one unbranched and seven (76) branched rays, inserted below 2^nd^ or 3^rd^ branched dorsal-fin ray; tip of adpressed reaching midway to anal-fin origin. Anal fin with three unbranched and six (76) branched rays; origin nearer to caudal-fin base than to pelvic-fin insertion. Caudal fin deeply forked, with nine branched rays on upper lobes and eight branched rays on lower lobes, lobes pointed.

Total vertebrae 4+34 (holotype). Gill rakers rudimentary. Pharyngeal teeth “5–5” (in one row). Air-bladder small, anterior chamber enveloped in thick fibrous capsule; posterior chamber small, elongated oval shaped, length less than half eye diameter (length 30.4%–34.9% of eye diameter), 9.2%–11.8% of head length.

##### Coloration in life.

Dorsal side of head and body yellowish grey, mid-lateral side shallow yellowish grey, and ventral side grayish white. Dorsal side of body with four distinct black crossbars (1^st^ and 2^nd^ at dorsal-fin base origin and ending respectively, 3^rd^ at vertical position above anal-fin base origin, 4^th^ on caudal peduncle respectively). Lateral side with seven or eight small black blotches; margin of scales above lateral line slightly black pigmented, lateral-line scales without obvious black spots, margin of first row below lateral line slightly black pigmented. One fluorescent green strip extend above lateral line. Interorbital region without black crossbar. Operculum and suborbital region with two distinct black blotches (one between anterior margin of eye and upper lip, the other expanded from posterior orbit to opercular) and one small black blotch exist between 2^nd^ and 3^rd^ suborbital plate. One black blotch above pectoral-fin base. Fins translucent, with small black pigments on some fin rays; dorsal-fin rays with some black spots; pectoral fin, pelvic fin with tiny black spots and lines; anal fin without spots; caudal-fin rays with two rows of black spots.

##### Coloration in preservation.

Dorsal side of head and body brownish yellow, mid-lateral side shallow brownish yellow, and ventral side grayish white. Dorsal side of body with four distinct black crossbars in same position as live individual. Lateral side with seven or eight small dark grey blotches; margin of scales above lateral line slightly black pigmented, lateral-line scales without obvious black spots, margin of first row below lateral line slightly black pigmented. The fluorescent green strip faded. Interorbital region without black crossbar. Operculum and suborbital region with two distinct black blotches in same position as live individual, and one small black blotch exist between 2^nd^ and 3^rd^ suborbital plate. One black blotch above pectoral-fin base. Fins pale, with small black pigments on some fin rays; dorsal-fin rays with some black spots; pectoral fin, pelvic fin with tiny black spots and lines; anal fin without spots; caudal-fin rays with two rows of black spots. The black pigments on fin rays faded after long-time preserve.

##### Sexual dimorphism.

No significant sexual dimorphism observed.

##### Distribution.

**Figure 4.**
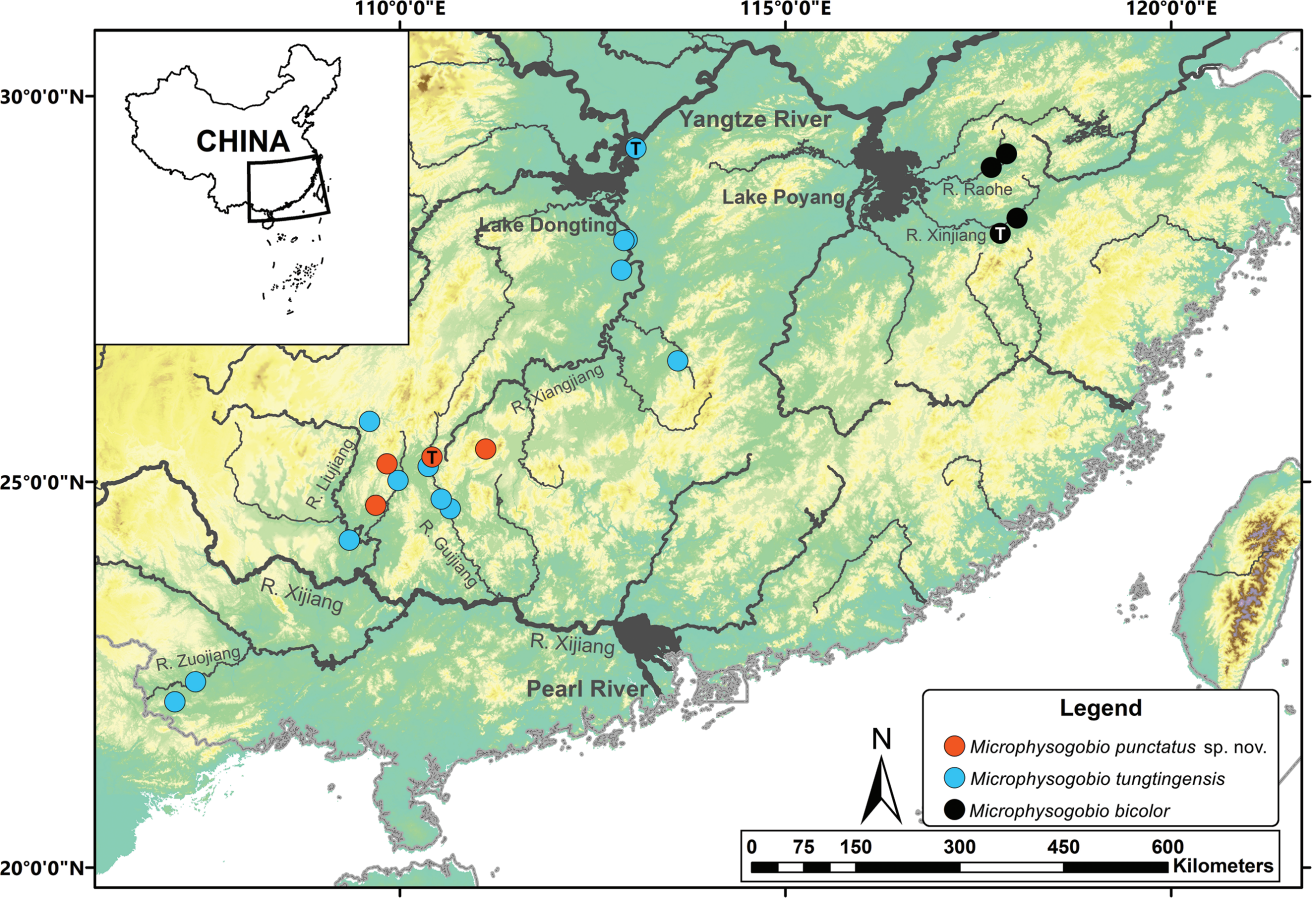
The distribution of the *Microphysogobiopunctatus* sp. nov., *M.tungtingensis* and *M.bicolor*. “T” in circles indicates the type locality of each species.

*Microphysogobiotungtingensis* is distributed in the Xiangjiang River system and the Lake Dongting of the middle Yangtze River basin. It is also found distributed in the Xijiang River system, which belongs to the Pearl River basin (Fig. 4).

##### Habitat and biology.

*Microphysogobiotungtingensis* inhabits the slow flowing water of rivers. It usually appears in areas with sandy bottoms with gravel and pebbles like other congeners. It can also be found in the water body with muddy bottom.

##### Etymology.

The name, *tungtingensis*, refers to its type locality, Lake Dongting. Chinese common name for this species is “洞庭小鳔鮈”.

#### ﻿ Microphysogobio punctatus sp. nov.

Taxon classificationAnimaliaCypriniformesCyprinidae

C2A2F1B4-4024-5F09-B5A1-FF423C4450EF

https://zoobank.org/F4C9B518-70FC-4A3B-928C-A305F9511A78

**Figure 5.**
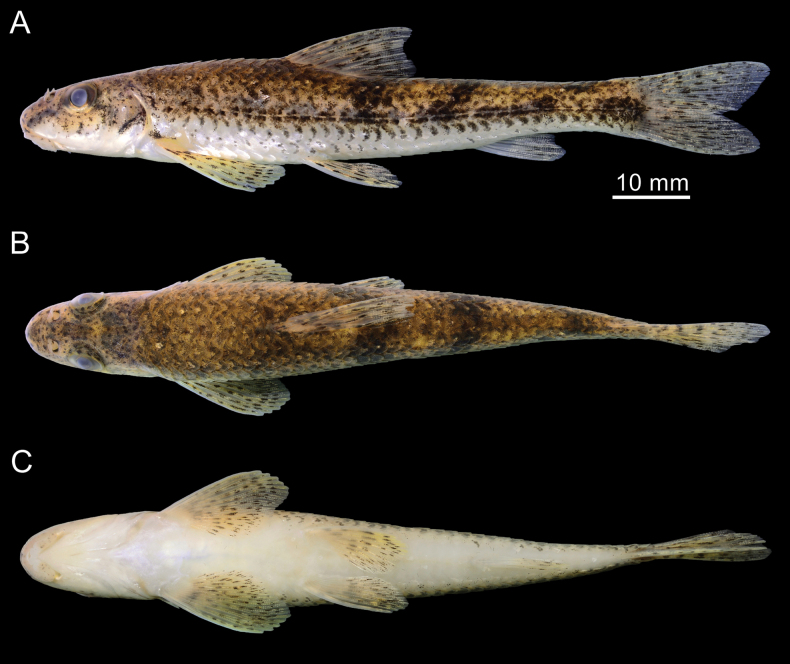
*Microphysogobiopunctatus* sp. nov., holotype, ASIZB 240329, 75.4 mm SL **A** lateral view **B** dorsal view **C** ventral view; photos were taken after fixed by formalin.

**Figure 6.**
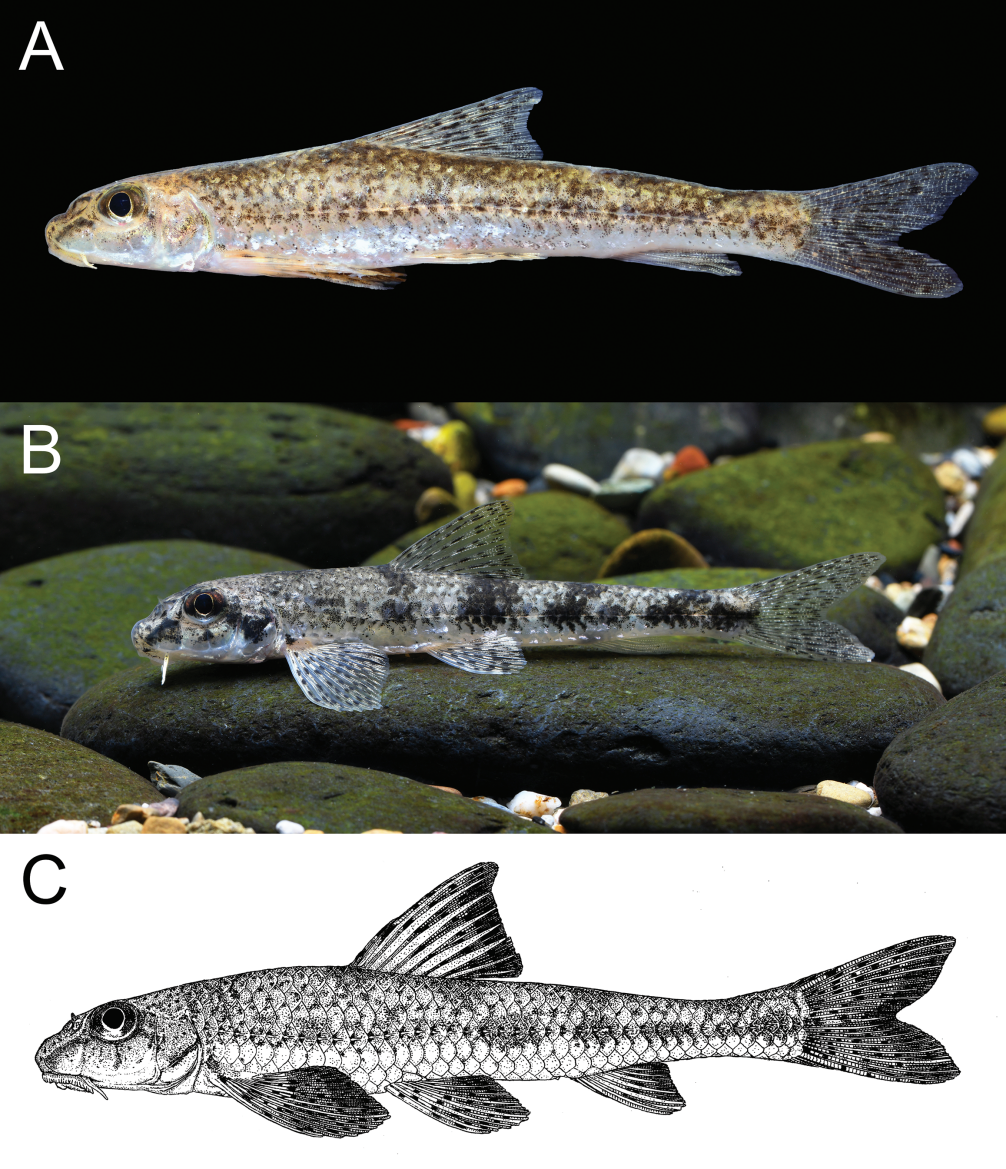
**A** Freshly caught *Microphysogobiopunctatus* sp. nov. from its type locality, uncatalogued **B** live individual from Luzhai County, Liuzhou City, Guangxi Autonomous Region in Luoqingjiang River, collected and photographed by Dr. Fan Li **C** original drawing by Zhi-Xian Sun based on individual collected from Yongfu County, Guilin City, Guangxi Autonomous Region in Xihe River.

Figs 5 , 6 ; Table 3

Microphysogobio kiatingensis : Wen (2006): 226.
Microphysogobio tungtingensis : Xiao and Lan (2023): 156.

##### Type material.

***Holotype*.** • ASIZB 240329, 75.4 mm SL; Lingchuan County, Guilin City, Guangxi Zhuang Autonomous Region, from the Huangshahe River, a tributary of the Lijiang River (25.3202379°N, 110.42292216°E, 166 m a.s.l.); collected by Zhixian Sun, Chen Tian, and Weihan Shao, 31 December 2023. ***Paratypes*.** • ASIZB 240330–5 (6 specimens), 240450–60 (11 specimens), 53.4–78.9 mm SL; IHB-T-A0000009, 1 specimen, 73.7 mm SL; SHOU 20231231061, 1 specimen, 73.8 mm SL; same collection information as holotype.

##### Additional material examined.

• ASIZB 240528–38, 11 specimens, 56.0–75.2 mm SL; Guanyang County, Guilin City, Guangxi Zhuang Autonomous Region, from the Guanjiang River, a tributary of the Xiangjiang River; collected by Zhixian Sun; 5 December 2020 • ASIZB 240505–10, 6 specimens, 53.9–73.1 mm SL; Guanyang County, Guilin City, Guangxi Zhuang Autonomous Region, from the Guanjiang River, a tributary of the Xiangjiang River; collected by Zhixian Sun; 20 January 2021 • ASIZB 240511–27, 17 specimens, 47.4–78.2 mm SL; Guanyang County, Guilin City, Guangxi Zhuang Autonomous Region, from the Guanjiang River, a tributary of the Xiangjiang River; collected by Zhixian Sun; 31 December 2021 • ASIZB 240539–42, 4 specimens, 58.1–70.7 mm SL; Yongfu County, Guilin City, Guangxi Zhuang Autonomous Region, from the Luoqingjiang River; collected by Junhao Huang and Zhuocheng Zhou; 27 November 2020 • ASIZB 240300–14, 240423–8, 21 specimens, 65.0–76.0 mm SL; IHB-YF-2024010201–3, 3 specimens, 71.3–76.9 mm SL; SOU 20240102121–3, 3 specimens, 69.2–74.0 mm SL; Shebian Village, Longjiang Township, Yongfu County, Guilin City, Guangxi Zhuang Autonomous Region, from the Xihe River, a tributary of the Luoqingjiang River (25.22972694°N, 109.83651085°E, 205 m a.s.l.); collected by Zhixian Sun, Chen Tian and Dong Sheng; 2 January 2024.

##### Diagnosis.

This new species can be distinguished from its congeners by a combination of the following characteristics: Posterior chamber of air-bladder small, globular or oval shaped, length 15.8%–26.4% of head length, and 58.6%–82.8% of eye diameter; upper jaw cutting-edge narrow, width less than half mouth width; caudal peduncle slender, depth 34.6%–48.5% of length; lateral-line scales 37–40 (mode 38, mean 39); circumpeduncular scales 12; branched anal-fin rays 6; midventral region of body scaleless only before pectoral-fin base end; all fins with numerous small black spots.

##### Description.

Body elongated, thoracic region flattened, abdomen rounded, caudal peduncle slender, compressed laterally. Dorsal body profile rising from nostrils to dorsal-fin origin, dropping along dorsal-fin base, then gradually sloping to caudal-fin base. Maximum body depth at dorsal-fin origin, body depth 14.6%–19.5% of standard length. Head elongated, length larger than body depth; snout blunt, with moderate concavity on top of snout before nostrils; eye diameter 30.7%–38.5% of head length, positioned at dorsal half of head; interorbital region flattened, width smaller than eye diameter (39.2%–67.9% of eye diameter). Anus positioned at anterior one-third of distance from pelvic-fin insertion to anal-fin origin.

**Table 3.** Morphometric measurements of *Microphysogobiopunctatus* sp. nov. and *M.bicolor*.

Characters	*Microphysogobiopunctatus* sp. nov. (*n* = 85)				*Microphysogobiobicolor* (*n* = 26)			
	Holotype	Holotype + Paratypes + Other Specimens			Holotype	Holotype + Other Specimens		
		Range	Mean	SD		Range	Mean	SD
Branched dorsal-fin rays	7	7	7		7	7	7	
Branched anal-fin rays	6	6	6		5	5	5	
Branched pectoral-fin rays	12	11–13	12		12	11–12	12	
Branched pelvic-fin rays	7	7	7		7	7	7	
Lateral line scales	37	37–40	39		37	36–38	37	
Scales above lateral line	3.5	3.5–4	3.5		3.5	3.5	3.5	
Scales below lateral line	2	2	2		2	2	2	
Pre-dorsal scales	10	9–11	10		9	9–10	10	
Circumpeduncular scales	12	12	12		12	12	12	
Standard Length (mm)	75.4	47.4–78.9			58.7	39.1–84.5		
**As percentage of SL**								
Body depth	16.4	14.6–19.5	17.1	1.0	19.0	15.7–24.3	19.0	2.2
Head length	22.1	20.7–25.4	23.1	0.9	22.1	22.1–26.1	23.8	0.8
Dorsal-fin length	23.3	21.9–28.2	24.4	1.4	22.9	20.6–24.7	22.6	1.1
Dorsal-fin base length	12.1	11.5–15.5	13.4	0.8	12.8	11.7–14.8	13.5	0.7
Pectoral-fin length	21.9	21.0–28.3	23.6	1.5	22.5	18.7–23.1	21.0	1.3
Pectoral-fin base length	5.2	4.4–6.4	5.4	0.4	5.3	4.8–6.4	5.5	0.4
Pelvic-fin length	17.9	16.2–20.8	18.6	0.9	18.0	14.3–18.1	16.4	1.0
Pelvic-fin base length	4.1	3.4–4.9	4.0	0.3	3.4	3.4–5.7	4.4	0.5
Anal-fin length	16.4	14.3–20.3	17.0	1.0	14.4	12.8–16.2	14.6	0.7
Anal-fin base length	6.6	6.1–8.9	7.3	0.5	5.3	5.3–7.6	6.8	0.5
Pre-dorsal length	42.6	40.4–46.3	43.2	1.1	44.4	41.2–46.1	43.8	1.2
Pre-pectoral length	23.2	21.9–26.8	24.1	0.8	23.6	23.6–29.7	26.4	1.5
Pre-pelvic length	45.7	44.0–51.2	47.5	1.3	46.5	46.5–58.4	52.1	3.3
Pre-anal length	73.9	71.5–78.1	74.7	1.5	77.3	75.5–90.0	81.0	3.3
Caudal peduncle length	18.5	16.2–21.0	18.4	0.9	17.5	15.8–19.3	17.3	1.0
Caudal peduncle depth	7.5	6.4–9.0	7.6	0.5	9.4	7.9–9.9	8.6	0.6
Head Length (mm)	16.7	10.6–19.2			13.0	9.1–22.0		
**As percentage of HL**								
Head depth	59.9	52.2–62.4	56.5	2.1	65.2	50.4–65.2	55.9	3.5
Head width	59.9	50.9–63.7	57.5	2.6	66.0	55.9–66.0	61.0	2.7
Eye diameter	31.8	30.7–38.5	33.6	1.7	31.5	26.0–33.9	30.2	1.9
Interorbital width	23.8	19.4–30.9	24.6	2.2	21.8	21.0–34.5	27.8	4.2
Snout length	35.6	31.7–43.2	37.6	2.5	32.4	32.4–43.4	38.0	2.5
Anterior papillae length	28.8	26.5–38.2	32.3	2.8	25.4	25.4–38.1	30.8	3.0
Anterior papillae width	28.3	17.6–36.1	27.2	4.1	22.5	21.8–39.9	29.8	4.6
Central anterior papillae width	9.8	4.3–11.0	6.7	1.4	4.9	4.4–11.0	6.2	1.5
Upper jaw cutting edge width	11.4	5.7–11.8	8.7	1.5	6.1	6.1–11.7	8.3	1.5
Medial pad width	13.5	9.6–19.4	12.9	1.5	10.1	9.7–14.1	11.7	1.2
Mouth depth	18.9	15.1–23.4	19.9	1.6	16.4	15.4–23.8	18.9	2.2
Mouth width	24.4	17.9–30.1	24.7	2.6	18.3	18.3–33.1	24.6	3.3
Barbel length	15.4	8.9–25.6	18.2	4.1	11.3	11.3–22.7	17.5	3.2

Mouth horseshoe-shaped and inferior, with one pair of maxillary barbels rooted at extremity of upper lip, barbel length shorter than eye diameter (26.4%–79.3% of eye diameter, 54.4% in average); upper and lower jaws with thin horny sheaths on cutting margins, upper jaw cutting edge width smaller than half mouth width (22.0%–46.8% of mouth width, 35.2% in average). Lips thick, well developed, with pearl-like papillae; central portion of anterior papillae arranged in one row, usually consists 2–6 papillae, tightly contact with each other (occasionally separated, Fig. 3C), size slightly larger than other lateral side papillae; lateral portions of anterior papillae in several rows; medial pad on lower lip heart-shaped, shallow grooved on surface; lateral lobes on lower lip covered with multiple developed papillae, posteriorly disconnected from each other behind medial pad and laterally connected with upper lip anterior papillae at mouth corner (Fig. 3C, D).

Body covered with moderately small cycloid scales. Lateral line complete, almost straight in lateral center, slightly bent down under dorsal origin. Lateral line scales 37 (6 specimens), 38 (38), 39 (29), 40 (12); scales above lateral line 3.5 (72), 4 (13); scales below lateral line 2 (85); pre-dorsal scales 9 (23), 10 (60), 11 (2); circumpeduncular scales 12 (85). Midventral region of body scaleless only before pectoral-fin base end.

Dorsal fin with three unbranched and seven (76 specimens) branched rays; distal margin slightly concave, origin nearer to snout than caudal-fin base. Pectoral fin with one unbranched and 11 (2), 12 (57), 13 (26) branched rays; tip of adpressed almost reaching or slightly extending anterior margin of pelvic-fin base. Pelvic fin with one unbranched and seven (85) branched rays, inserted below 2^nd^ or 3^rd^ branched dorsal-fin ray; tip of adpressed reaching or extending midway to anal-fin origin. Anal fin with three unbranched and six (85) branched rays; origin almost equal-distant from caudal-fin base to pelvic-fin insertion. Caudal fin deeply forked, with nine branched rays on upper lobes and eight branched rays on lower lobes, lobes pointed.

Gill rakers rudimentary. Pharyngeal teeth “5–5” (in one row). Air-bladder small, anterior chamber enveloped in thick fibrous capsule; posterior chamber small, globular or oval shaped, length less than eye diameter (58.6%–82.8%, mean 68.2%), 14.6%–26.4% (mean 22.8%) of head length.

##### Coloration in life.

Dorsal side of head and body brownish grey, mid-lateral side shallow brownish grey, and ventral side grayish white. Dorsal side of body with five distinct black crossbars (1^st^ on back of nape, 2^nd^ and 3^rd^ at dorsal-fin base origin and ending respectively, 4^th^ at vertical position above anal-fin base origin, 5^th^ on caudal peduncle respectively). Lateral side with 8–10 different sized black blotches, some blotches sometime connect with the 3^rd^, 4^th^, and 5^th^ crossbar on the lateral side of body; scales above lateral line black pigmented, lateral-line scales with obvious black spots, margin of first row below lateral line black pigmented, and 2^nd^ row below lateral line slightly black pigmented. Interorbital region with a black crossbar. Operculum and suborbital region with two distinct black blotches (one between anterior margin of eye and upper lip, the other expanded from posterior orbit to opercular) and one small black blotch exist between 2^nd^ and 3^rd^ suborbital plate. One black blotch above pectoral-fin base. Fins membrane translucent, with numerous black pigments on some fin rays; dorsal-fin rays glittery green, with many black spots and dash lines; pectoral fin rays and pelvic fin rays glittery green, with many black spots and dash lines; anal fin rays with some black spots; caudal-fin rays with numerous black spots (Fig. 6A, B).

##### Coloration in preservation.

Dorsal side of head and body brownish yellow, mid-lateral side shallow brownish yellow, and ventral side grayish white. Dorsal side of body with five distinct black crossbars in same position as live individual. Lateral side with 8–10 differently sized dark grey blotches; scales above lateral line black pigmented, lateral-line scales without obvious black spots, margin of first row below lateral line slightly black pigmented. Interorbital region with a black crossbar. Operculum and suborbital region with two distinct black blotches in same position as live individual, and one small black blotch exist between 2^nd^ and 3^rd^ suborbital plate. One black blotch above pectoral-fin base. Fins membrane pale, with numerous black pigments on some fin rays in same position as live individual, glittery green on fin rays faded.

##### Sexual dimorphism.

No significant sexual dimorphism observed.

##### Distribution.

According to the field collections, this species is distributed in the Guijiang and Liujiang rivers, two northern tributaries of the Xijiang River system, which belongs to the Pearl River basin. It is also found in the upper Xiangjiang River, which drains into the middle Yangtze River basin (Fig. 4).

##### Habitat and biology.

*Microphysogobiopunctatus* sp. nov. inhabits the slow flowing water of rivers approximately 30–40 meters wide. It usually appears in areas with sandy bottoms with gravel and pebbles. Co-exiting species includes e.g., *Opsariichthysbidens*, *Acheilognathustonkinensis*, *Squalidusargentatus*, *Sarcocheilichthys* sp. *Microphysogobiozhangi*, and *Cobitis* spp.

##### Etymology.

The new species name *punctatus* is derived from the Latin *punctum*, meaning spot. The name refers to the numerous black spots on its scales and fin rays. Suggested Chinese name for this species is “斑点小鳔鮈”.

## ﻿Discussion

### ﻿*Microphysogobioelongatus*, a junior synonym of *M.tungtingensis*

*Microphysogobioelongatus* was originally described as a slender (body depth less than 16.7% of SL) species with six branched anal-fin rays and distribution in the Xijiang River, the tributary of the Pearl River basin (Luo et al. 1977). In its original description, it can be distinguished from “*M.tungtingensis*” (based on three non-types) only by their branched anal-fin rays (six rays in *M.elongatus* vs five rays in “*M.tungtingensis*”) and their distribution. According to the subsequent studies which followed Luo et al. (1977), the key character between *M.tungtingensis* and *M.elongatus* is still basically on branched anal-fin ray count. However, after carefully checking the original descriptions of *M.tungtingensis* along with the other *Microphysogobio* species described by Nichols himself (Nichols 1926a, 1926b, 1930) and examining the X-ray photos of those types, we confirmed that the branched anal-fin rays of *M.tungtingensis* should be six. Thus, it is not easy to distinguish *M.tungtingensis* and *M.elongatus* in this situation but only by their distribution (former in the Lake Dongting system in the middle Yangtze River basin vs latter in the Xijiang River system of the Pearl River basin), causing the validity of the latter suspicious.

**Figure 7.**
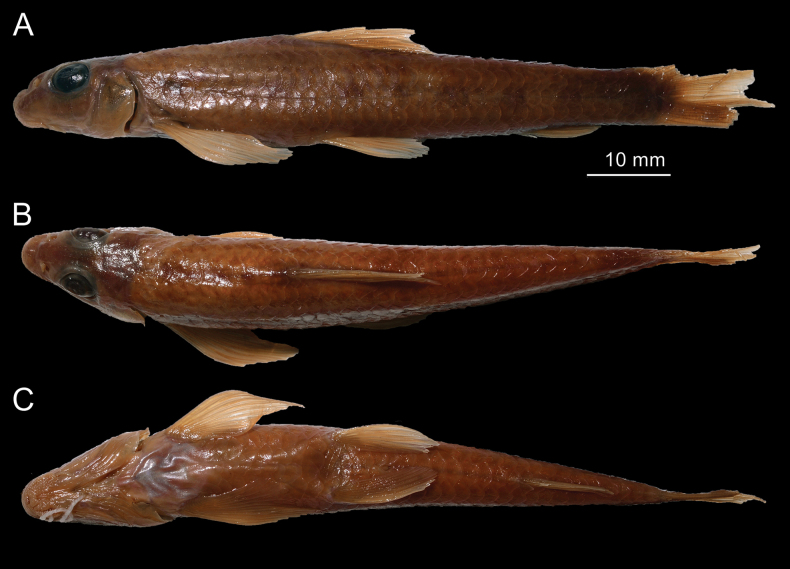
*Microphysogobioelongatus* (= *M.tungtingensis*), syntype, IHB 58-7-876, 77.2 mm SL **A** lateral view **B** dorsal view, and **C** ventral view.

**Figure 8.**
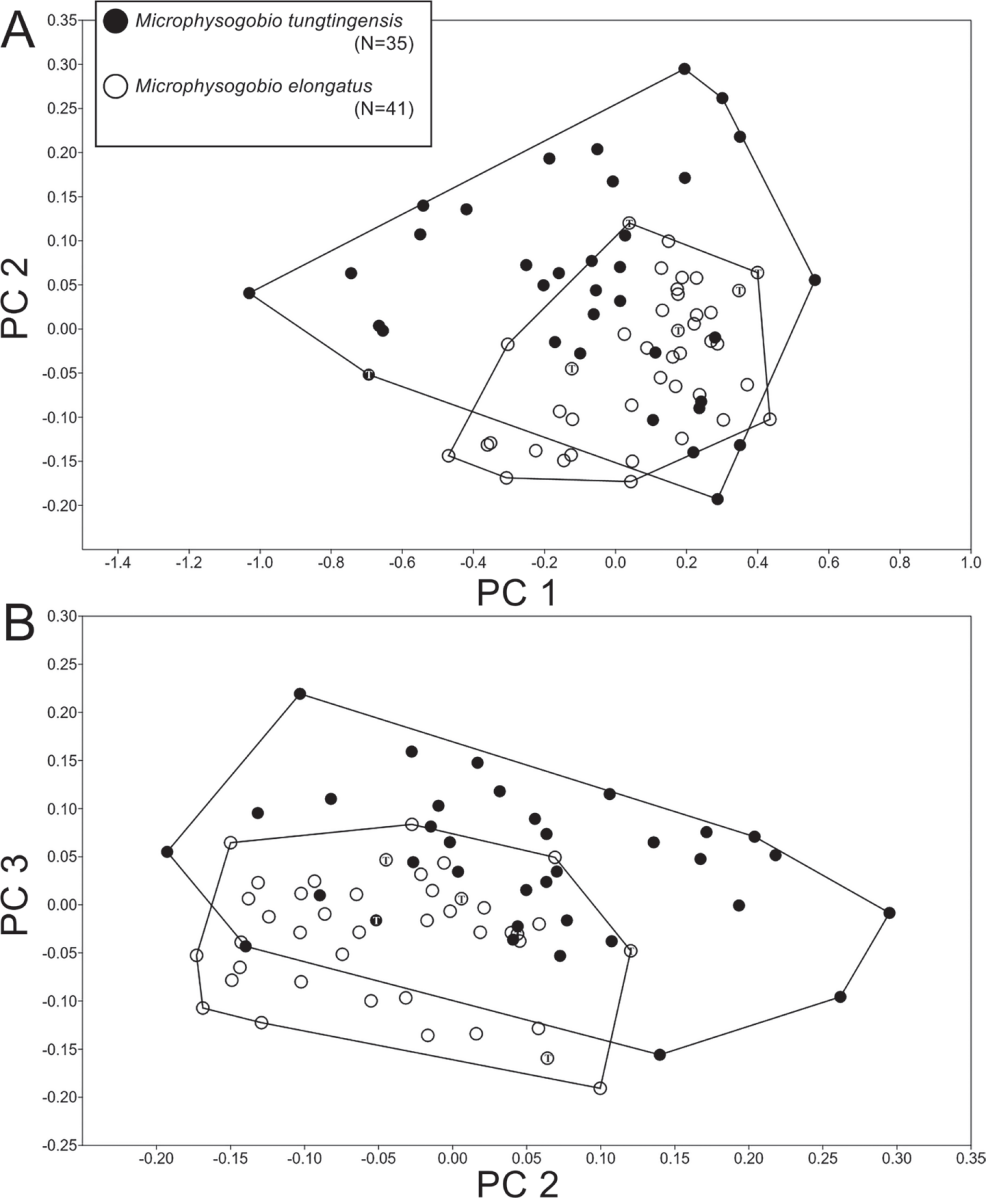
Scatterplot of the PC1 against PC2 (**A**) and PC2 against PC3 (**B**) extracted from 30 morphometric data of *Microphysogobiotungtingensis* and *M.elongatus*. Letter “T” marked inside the dots indicate the type specimens.

In order to understand the potential morphological differences between these two species, 76 specimens, including the types of both species, from a wide geographical range, were examined (Figs 1, 7). The result shows that there is no significant difference in both counts and measurable traits (Table 2). The PCA on those morphometric measurements were performed to make comparisons in detail. The first three component contributed 82.3% of the variance. Principle component 1 (PC 1) represents most on the body size of the specimens, while PC 2 and PC 3 reflected their morphology. The PCA loadings are presented in Table 4, showing that the lip papillae system pattern contributes most in PC 2 and PC 3. However, both PC1 against PC2 and PC2 against PC3 are not able to separate these two species (Fig. 8).

**Table 4.** Loadings on the first three principal components extracted from morphometric data of *Microphysogobiotungtingensis* and *M.elongatus*.

Morphometric measurements	PC 1	PC 2	PC 3
Standard length	0.171	-0.073	0.058
Body depth	0.212	-0.108	0.213
Head length	0.181	-0.015	0.039
Head depth	0.187	-0.039	0.112
Head width	0.192	-0.017	0.135
Dorsal-fin length	0.161	-0.135	-0.009
Dorsal-fin base length	0.184	-0.106	0.119
Pectoral-fin length	0.174	-0.135	-0.043
Pectoral-fin base length	0.190	-0.073	0.112
Pelvic-fin length	0.165	-0.168	-0.048
Pelvic-fin base length	0.189	-0.114	0.210
Anal-fin length	0.168	-0.155	-0.038
Anal-fin base length	0.149	-0.122	0.033
Caudal peduncle length	0.170	-0.145	0.082
Caudal peduncle depth	0.195	-0.137	0.085
Eye diameter	0.169	-0.053	-0.034
Interorbital width	0.185	-0.086	0.012
Snout length	0.202	0.115	0.180
Pre-dorsal length	0.180	-0.040	0.052
Pre-pectoral length	0.175	-0.018	0.044
Pre-pelvic length	0.177	-0.058	0.088
Pre-anal length	0.171	-0.071	0.032
Anterior papillae length	0.229	0.115	-0.171
Anterior papillae width	0.161	**0.699**	**0.343**
Central anterior papillae width	0.155	0.105	-**0.414**
Upper jaw cutting edge width	0.117	**0.342**	-0.181
Medial pad width	0.224	0.087	-**0.322**
Mouth depth	0.237	0.160	-0.112
Mouth width	0.175	**0.332**	-0.017
Barbel length	0.187	-0.022	-**0.559**

**Figure 9.**
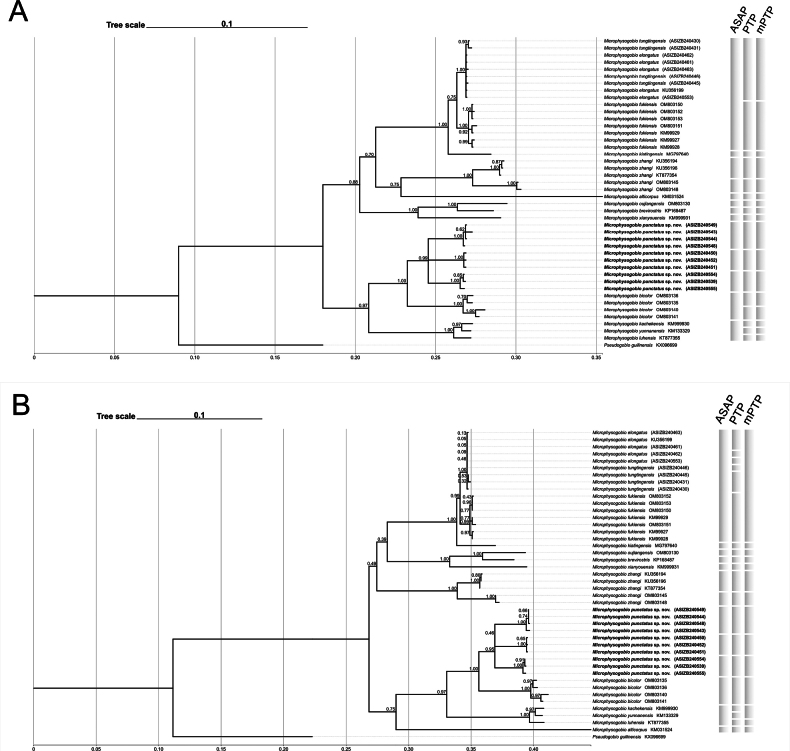
Molecular phylogenetic trees of *Microphysogobiopunctatus* sp. nov., *M.tungtingensis*, *M.elongatus* and other phylogenetically close related congeners based on Cyt *b* sequence (1140 bp) **A** Bayesian Inference method **B** maximum likelihood method.

Molecular phylogenetic analyses were also conducted. The intraspecific distance of *M.tungtingensis* and *M.elongatus* was 0.16% and 0.04% respectively, while the interspecific distance between them is 0.13%. This value is approximate to the intraspecific distance of *M.tungtingensis*, making it hard to tell they are different. Besides, the interspecific distance is far less than any other showed in the genus *Microphysogobio*. The phylogenetic trees (Fig. 9) also demonstrate undetectable length of the branches.

Although distributed in different river basins, such distribution might be interpreted by two previously suggested hypotheses. One is that the Lingqu Canal, an ancient artificial canal connected in 214 B.C. during Qin Dynasty which connecting the Xiangjiang River with the Lijiang River, might cause the trans-basin gene flow between the Yangtze River basin and the Pearl River basin, which was discovered for *M.zhangi* (Huang et al. 2017). This interpretation was supported by the shared haplotype (Table 1) from both river basins. The second hypothesis is the episodic river capture events between the interwoven watersheds of the Nanling Mountains’ complex landform, e.g., the Lijiang River capture (Ru 2011), causing the cross-basin distribution, which was found in *Sarcocheilichthysparvus* (Li et al. 2023). Given the low intraspecific and interspecific distances (Table 5) within the phylogenetic linage *M.tungtingensis* located (*M.tungtingensis* + *M.elongatus* + *M.fukiensis* + *M.kiatingensis*) and their broad geographical distribution (from the upper Yangtze River basin to the southeast coastal river systems), we drew the conclusion that the species within this linage performed a relatively conserved mutation in molecular. Hence, according to the trees shown in Fig. 9, it is also likely that the cross-basin distribution was caused by the very recent river capture event which was not long enough ago to accumulate a certain mutation to be detected by the current mitochondrial gene data, especially in this conserved linage.

**Table 5.** Genetic distances of the Cyt *b* gene computed by MEGA 6.0 amongst 14 analyzed species of *MicrophysogobioI*; *Pseudogobioguilinensis* was used as the outgroup.

		Intraspecific	1	2	3	4	5	6	7	8	9	10	11	12	13	14
1	*Microphysogobiopunctatus* sp. nov.	0.0324														
2	*M.bicolor*	0.0123	0.0665													
3	*M.oujiangensis*	NA	0.1335	0.1314												
4	*M.brevirostris*	NA	0.1291	0.1328	0.0496											
5	*M.xianyouensis*	NA	0.1392	0.1377	0.0905	0.0834										
6	*M.alticorpus*	NA	0.1345	0.1407	0.1555	0.1604	0.1464									
7	*M.zhangi*	0.0263	0.1303	0.1365	0.1331	0.1218	0.1283	0.1386								
8	*M.kiatingensis*	NA	0.1290	0.1353	0.1250	0.1230	0.1106	0.1476	0.1083							
9	*M.elongatus*	0.0004	0.1206	0.1299	0.1061	0.1116	0.1102	0.1381	0.1080	0.0336						
10	*M.tungtingensis*	0.0016	0.1203	0.1290	0.1062	0.1128	0.1113	0.1388	0.1082	0.0346	0.0013					
11	*M.fukiensis*	0.0037	0.1234	0.1291	0.1106	0.1151	0.1113	0.1367	0.1068	0.0364	0.0150	0.0155				
12	*M.kachekensis*	NA	0.0980	0.1011	0.1298	0.1255	0.1298	0.1392	0.1316	0.1373	0.1289	0.1289	0.1299			
13	*M.luhensis*	NA	0.0947	0.1026	0.1330	0.1254	0.1341	0.1360	0.1314	0.1408	0.1279	0.1279	0.1306	0.0205		
14	*M.yunnanensis*	NA	0.0961	0.1023	0.1274	0.1232	0.1318	0.1339	0.1315	0.1396	0.1289	0.1289	0.1288	0.0115	0.0196	
15	*P.guilinensis* (outgroup)	NA	0.1678	0.1665	0.1686	0.1629	0.1658	0.1859	0.1634	0.1548	0.1482	0.1489	0.1528	0.1570	0.1594	0.1593

Based on the morphological comparisons, molecular phylogenetic analyses, and its distribution, we suggest *M.elongatus* to be a junior synonym of *M.tungtingensis*. On the basis of the revision of *M.tungtingensis*, we are now able to distinguish the new species, with an overlapping distribution, discussed below.

### ﻿Morphological comparison of the new species

Among the 30 known *Microphysogobio* species, *M.punctatus* sp. nov. can be distinguished from *M.brevirostris* (Günther, 1868), *M.chinssuensis* (Nichols, 1926), *M.yaluensis* (Mori, 1928), *M.hsinglungshanensis* Mori, 1934, *M.koreensis* Mori, 1935, *M.longidorsalis* Mori, 1935, *M.amurensis* (Taranetz, 1937), *M.alticorpus* Bănărescu & Nalbant, 1968, *M.anudarini* Holcík & Pivnicka, 1969, *M.liaohensis* (Qin, 1987), *M.rapidus* Chae & Yang, 1999, *M.jeoni* Kim & Yang, 1999, *M.wulonghensis* Xing, Zhao, Tang & Zhang, 2011, *M.nudiventris* Jiang, Gao & Zhang, 2012, *M.xianyouensis* Huang, Chen & Shao, 2016, and *M.oujiangensis* Sun & Zhao, 2022 by having a narrower upper jaw cutting edge (width 22.0%–46.8% of mouth width vs larger than half of mouth width).

For the remaining 14 congeners, the new species can be distinguished from *M.linghensis* Xie, 1986 and *M.microstomus* Yue, 1998 by having well developed lip papillae system (vs undeveloped or less developed papillae on lips, Fig. 3E). It can be distinguished from *M.kiatingensis* (Wu, 1930) by having a larger squamous zone on mid-ventral region (scaleless only before pectoral-fin base end vs scaleless before two-thirds of the distance from the pectoral-fin insertion to the pelvic-fin insertion). It can be distinguished from *M.bicolor* (Nichols, 1930), *M.tafangensis* (Wang, 1935), *M.zhangi* Huang, Zhao, Chen & Shao, 2017 by having more branched anal-fin rays (6 vs 5). For *M.zhangi*, the species sympatric with the new species, it can be further distinguished from the new species by having different anterior papillae pattern on upper lip (two large papillae vs two to six similar sized papillae, Fig. 3D, F) It can also be distinguished from *M.fukiensis* (Nichols, 1926), *M.tungtingensis* (Nichols, 1926) and *M.vietnamica* Mai, 1978 by having larger posterior chamber of the air-bladder (length 58.6%–82.8% of eye diameter vs length 21.9%–43.1%, 30.4%–34.9% and 35.5%–64.2% of eye diameter respectively). Considering *M.vietnamica* has overlap on air-bladder size with the new species, it can be further distinguished from latter by having different anterior papillae pattern on upper lip (two large papillae separated from each other vs similar sized papillae tightly contacted with each other, Fig. 3D, G). The new species can be distinguished from *M.pseudoelongatus* Zhang & Zhao, 2001 by having more circumpeduncular scales (12 vs 10). For the *M.nikolskii* (Dao & Mai, 1959), the species inquirenda (Kottelat 2013), the new species can be distinguished from it by having fewer lateral-line scales (37–40 vs 43; Huang et al. 2018).

For the rest of the three congeners, they are morphologically similar with the new species by having a larger posterior chamber of the air-bladder (larger than half eye diameter and sometimes equal to eye diameter) and tightly contacted central portion of anterior papillae. The new species can be distinguished from these three species by having more black pigmentation on fin rays. In addition, the new species can be distinguished from *Microphysogobioyunnanensis* (Yao & Yang, 1977) by having fewer scales above lateral line (3.5–4 vs 4.5), from *M.luhensis* Huang, Chen, Zhao & Shao, 2018 by having a larger eye diameter (30.7%–38.5% vs 25.2%–27.9% of head length), and from *M.kachekensis* (Oshima, 1926) by having a more slender caudal peduncle (depth 34.6%–48.5% vs 48.9%–55.7% of length).

**Figure 10.**
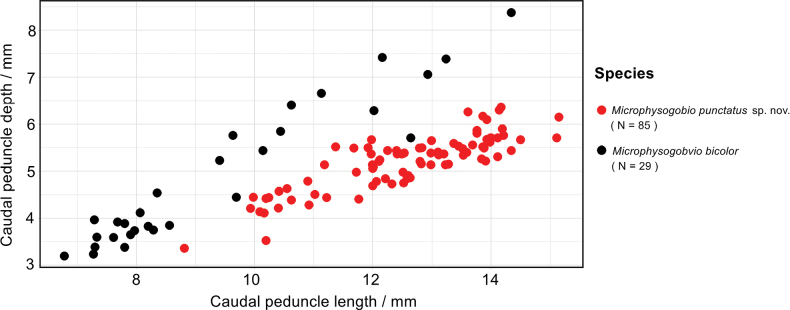
Morphometric measurement divergence in caudal peduncle depth between *Microphysogobiopunctatus* sp. nov. and *M.bicolor*.

As for *Microphysogobiobicolor*, although it has already been distinguished from the new species by having fewer branched anal-fin rays (5 vs 6), it is also morphologically close to the new species by having similar size of the air-bladder (63.1%–82.0% vs 50.3%–82.8% of eye diameter in average) and lip papillae pattern (Fig. 3H). However, the new species has a slender caudal peduncle (depth 34.6%–48.5% vs 43.3%–58.4% of length; Fig. 10) and shorter preanal distance (71.5%–78.1% vs 75.5%–90.0% of SL) as compared to *M.bicolor*.

A total of 37 haplotypes from 43 *Microphysogobio* individuals for Cyt *b* gene were included in the molecular phylogeny study. Based on molecular phylogenetic analyses, the new species is sister to *M.bicolor*, and together sister to *M.luhensis*–*M.kachekensis*–*M.yunnanensis* clade. The interspecific distance between *M.punctatus* and *M.bicolor* is 6.65% for Cyt *b* based on K2P model. The intraspecific distance within *M.punctatus* is 3.24% based on K2P model, which is lower than the interspecific distance. In fact, the molecular species delineation method (ASAP and PTP) inferred that the three geographic populations (Lingchuan, Yongfu, and Guanyang counties) to be three different molecular species which forming a monophyletic lineage. However, there is no significant difference in morphology among three populations, plus the closely connected river system within their distribution, we treated them as one species.

### ﻿Comparative material

***Microphysogobiobicolor***: • AMNH 9678, holotype, 58.7 mm SL; Hekou Township, Yanshan County, Shangrao City, Jiangxi Province, from the Xinjiang River; collected by Clifford H. Pope; 22 June 1926 • ASIZB 220619–27, 9 specimens, 39.1–50.3 mm SL; Yongping Township, Yanshan County, Shangrao City, Jiangxi Province, from the Yanshanhe River (28.21615237°N, 117.79050896°E, 75 m a.s.l.); collected by Zhixian Sun and Rui Zhang; 11–12 April 2021 • ASIZB 213554, 1 specimen, 52.4 mm SL; Yongping Township, Yanshan County, Shangrao City, Jiangxi Province, from the Yanshanhe River (28.21615237°N, 117.79050896°E, 75 m a.s.l.); collected by Zhixian Sun, Rui Zhang, Chen Tian, and Xin Wang; 20 July 2021 • ASIZB 220628–35, 8 specimens, 42.1–62.2 mm SL; Taibai Township, Wuyuan County, Shangrao City, Jiangxi Province, from the Le’anhe River (29.07164455°N, 117.67277022°E, 47 m a.s.l.); collected by Junhao Li and Zhixian Sun; 16 December 2020 • ASIZB 220636–40, 220647, 220650, 220652, 220654, 9 specimens, 63.1–84.5 mm SL; bought from the Dongmen Market from the local fishermen, Wuyuan County, Shangrao City, Jiangxi Province, from the Le’anhe River; collected by Zhixian Sun and Rui Zhang; 11 April 2020 • ASIZB 229848–9, 2 specimens, 59.0–76.0 mm SL; bought from the Wangjiaba Market from the local fishermen, Guangxin District, Shangrao City, Jiangxi Province, from the Xinjiang River; collected by Zhixian Sun, Rui Zhang, Chen Tian, and Xin Wang; 21 July 2021.

***Microphysogobiofukiensis***: • ASIZB 220660–68, 9 specimens, 50.5–64.6 mm SL; bought from the local market, Guangze County, Nanping City, Fujian Province, from the Futunxi River; collected by Zhixian Sun, Rui Zhang, Qiuju Chen and Rui Xi; 17 April 2021.

***Microphysogobiokechekensis***: • ASIZB 240478–88, 11 specimens, 64.8–83.6 mm SL; bought from the local market, Baisha Li Autonomous County, Hainan Province, probably caught by fishermen from the Wanquanhe River; collected by Shanyu Wu; 3 April 2020 • ASIZB 69768, 1 specimen, 100.0 mm SL; Gongzheng Township, Shangsi County, Fangchenggang City, Guangxi Zhuang Autonomous Region, from a stream of Maolingjiang River; collected by Yahui Zhao; 29 April 1999.

***Microphysogobioluhensis***: • ASIZB 204717, paratype, 55.5 mm SL; Dongkeng Township, Luhe County, Shanwei City, Guangdong Province, from the Rongjiang River (23.30165309°N, 115.71898652°E, 126 m a.s.l.); collected by Shih-Pin Huang; 2 April 2009.

***Microphysogobiomicrostomus***: • SHOU 20231209017, 47.3 mm SL; Shanhu Township, Shengzhou City, Shaoxing City, Zhejiang Province, from the Shanxi River (29.63724853°N, 120.84212848°E, 12 m a.s.l.); collected by Zhixian Sun; 9 December 2023.

***Microphysogobiovietnamica***: • ASIZB 240561–71, 11 specimens, 43.0–53.5 mm SL; Shixing County, Shaoguan City, Guangdong Province, from the Mojianghe River; collected by Zhixian Sun; 4 January 2021.

***Microphysogobioyunnanensis***: • IHB 645480, 645486–7, 645492, 645494–5, 645499, syntypes, 7 specimens, 73.8–82.2 mm SL; Yuanjiang Hani Yi and Dai Autonomous County, Yuxi City, Yunnan Province, from the Yuanjiang River (the Red River); May 1964 • IHB 645496, 6440132, 6440136, 6440296, 6440638, 6440648–9, 6440654, 6450063, syntypes, 9 specimens, 68.0–95.9 mm SL; Hekou Yao Autonomous County, Honghe Hani and Yi Autonomous Prefecture, Yunnan Province, from the Yuanjiang River (the Red River); April and May 1964.

***Microphysogobiozhangi***: • ASIZB 204677, holotype, 75.1 mm SL; Quanzhou County, Guilin City, Guangxi Zhuang Autonomous Region, from the Xiangjiang River (25.93332646N, 111.08037923E, 151 m a.s.l.); collected by Shih-Pin Huang and Jinqing Huang; 6 November 2015 • ASIZB 220669–76, 8 specimens, 48.2–63.6 mm SL; Pingle County, Guilin City, Guangxi Zhuang Autonomous Region, from the Lijiang River; collected by Zhixian Sun; 23 January 2021.

## Supplementary Material

XML Treatment for Microphysogobio tungtingensis

XML Treatment for Microphysogobio punctatus

## ﻿Additional information

### Author ORCIDs

Zhi-Xian Sun https://orcid.org/0000-0003-0565-2801

Wen-Qiao Tang https://orcid.org/0000-0001-5992-5022

Ya-Hui Zha https://orcid.org/0000-0002-4615-596X

### Data availability

All of the data that support the findings of this study are available in the main text or Supplementary Information.

## Supplementary materials

Supplementary material 1 Raw data of the type specimens of *Microphysogobiotungtingensis* and *M.elongatus* Data type xlsx https://binary.pensoft.net/file/1148395This dataset is made available under the Open Database License (http://opendatacommons.org/licenses/odbl/1.0/). The Open Database License (ODbL) is a license agreement intended to allow users to freely share, modify, and use this Dataset while maintaining this same freedom for others, provided that the original source and author(s) are credited.Zhi-Xian Sun, Wen-Qiao Tang, Ya-Hui Zhao
